# Supplementary material for: End Sequence Analysis Toolkit (ESAT) expands the extractable information from single-cell RNA-seq data
Source: Genome Res. 2016 Oct;26(10):1397–410. doi: 10.1101/gr.207902.116 (PMC5052061; doi:10.1101/gr.207902.116)
Supplement: Supplemental Material [file supp_gr.207902.116_Supp_Materials.tar.gz › ESAT-esat_v0.1/doc/html/classbroad_1_1core_1_1siphy_1_1_stationary_distribution_i_o.html]

ESAT: broad.core.siphy.StationaryDistributionIO Class Reference


ESAT


- Main Page
- Related Pages
- Packages
- Classes
- Files

- Class List
- Class Index
- Class Hierarchy
- Class Members

All Classes Namespaces Files Functions Variables Enumerator Pages

- broad
- core
- siphy
- StationaryDistributionIO

Public Member Functions |
List of all members

broad.core.siphy.StationaryDistributionIO Class Reference

Collaboration diagram for broad.core.siphy.StationaryDistributionIO:

[legend]

|  |  |
| --- | --- |
| Public Member Functions | |
|  | StationaryDistributionIO () |
|  | |
| void | loadIntoDistribution (File source, EmpiricalDistribution ed, boolean shortFormat) throws IOException |
|  | |
| void | load (InputStream is) throws IOException |
|  | |
| void | load (String filePath) throws IOException |
|  | |
| void | shift (int amountToShift) |
|  | |
| List< PiFit > | getFits () |
|  | |
| Iterator< PiFit > | getOverlappers (LightweightGenomicAnnotation annotation) |
|  | |

## Constructor & Destructor Documentation

|  |  |  |  |  |
| --- | --- | --- | --- | --- |
| broad.core.siphy.StationaryDistributionIO.StationaryDistributionIO | ( |  | ) |  |

## Member Function Documentation

|  |  |  |  |  |
| --- | --- | --- | --- | --- |
| List<PiFit> broad.core.siphy.StationaryDistributionIO.getFits | ( |  | ) |  |

Here is the caller graph for this function:

|  |  |  |  |  |  |
| --- | --- | --- | --- | --- | --- |
| Iterator<PiFit> broad.core.siphy.StationaryDistributionIO.getOverlappers | ( | LightweightGenomicAnnotation | *annotation* | ) |  |

Here is the call graph for this function:

|  |  |  |  |  |  |
| --- | --- | --- | --- | --- | --- |
| void broad.core.siphy.StationaryDistributionIO.load | ( | InputStream | *is* | ) | throws IOException |

Here is the caller graph for this function:

|  |  |  |  |  |  |
| --- | --- | --- | --- | --- | --- |
| void broad.core.siphy.StationaryDistributionIO.load | ( | String | *filePath* | ) | throws IOException |

Here is the call graph for this function:

|  |  |  |  |
| --- | --- | --- | --- |
| void broad.core.siphy.StationaryDistributionIO.loadIntoDistribution | ( | File | *source*, |
|  |  | EmpiricalDistribution | *ed*, |
|  |  | boolean | *shortFormat* |
|  | ) |  | throws IOException |

Here is the call graph for this function:

|  |  |  |  |  |  |
| --- | --- | --- | --- | --- | --- |
| void broad.core.siphy.StationaryDistributionIO.shift | ( | int | *amountToShift* | ) |  |

---

The documentation for this class was generated from the following file:

- java/broad/core/siphy/StationaryDistributionIO.java


---

Generated on Fri Aug 15 2014 15:10:57 for ESAT by  

 1.8.7
